# Renal Vein Injury During Percutaneous Nephrolithotomy Procedure

**DOI:** 10.1089/cren.2016.0089

**Published:** 2016-09-01

**Authors:** Sarwar Noori Mahmood, Hewa Mahmood Toffeq

**Affiliations:** ^1^ Department of Surgery, School of Medicine, Faculty of Medical Sciences, University of Sulaimani, Sulaymaniyah, Iraq.; ^2^ Department of Urology, Sulaymaniyah General Teaching Hospital, Sulaymaniyah, Iraq.

**Keywords:** percutaneous nephrostolithotomy, renal vein injury

## Abstract

***Background:*** Percutaneous nephrostolithotomy is an important approach for removing kidney stones. Puncturing and dilatation are two mandatory steps in percutaneous nephrolithotomy (PCNL). Uncommonly, during dilatation, the dilators can cause direct injury to the main renal vein or to their tributaries.

***Case Presentation:*** A 75-year-old female underwent PCNL for partial staghorn stone in the left kidney. During puncturing and dilatation, renal vein tributary was injured, and the nephroscope entered the renal vein and inferior vena cava, which was clearly recognized. Injection of contrast material through the nephroscope confirms the false pathway to the great veins (renal vein and inferior vena cava). Bleeding was controlled intraoperatively by applying Amplatz sheath over the abnormal tract, the procedure was continued and stones were removed. At the end of the procedure, a Foley catheter was used as a nephrostomy tube and its balloon was inflated inside the renal pelvis and pulled back with light pressure to the lower calix, which was the site of injury to the renal vein tributaries, then the nephrostomy tube was closed; by this we effectively controlled the bleeding. The patient remained hemodynamically stable; antegrade pyelography was done on the second postoperative day, there was distally patent ureter with no extravasation, neither contrast leak to renal vein, and was discharged home at third postoperative day. After 2 weeks, the nephrostomy tube was gradually removed in the operative room, without bleeding, on the next day, Double-J stent was removed.

***Conclusion:*** Direct injury and false tract to the renal vein tributaries during PCNL can result in massive hemorrhage, and can be treated conservatively in hemodynamically stable patients, using a nephrostomy catheter as a tamponade.

## Background

Percutaneous nephrolithotomy (PCNL) was introduced by Ferström and Johansson in 1976, and it has remained an indispensable approach for kidney stones removal since its inception.^1^ Injuries to the main renal vessels are uncommon, accounting for less than 0.5%.^2^ Bleeding is mainly venous during percutaneous procedures and is usually mild and resolves spontaneously or responds to simple maneuvers such as placement of a large caliber nephrostomy tube into the tract.^3^ Severe bleeding complications of percutaneous renal surgery are commonly arterial in nature.^4,5^ Hemorrhage caused by pseudoaneurysms usually occurs in the postoperative period and can be managed best by selective angiographic embolization. Occasionally, nephrectomy is required for refractory bleeding.^5^

We reported one case of renal vein tributary injury during PCNL.

## Case Presentation

**FIG. 1.**
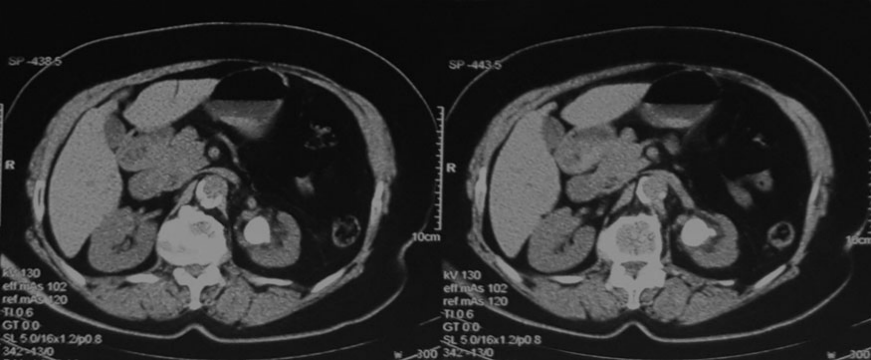
CT cross section slides of a 75-year-old female patient, showing large UPJ stone, thinning of left renal cortex, and renal pelvis dilation.

**FIG. 2.**
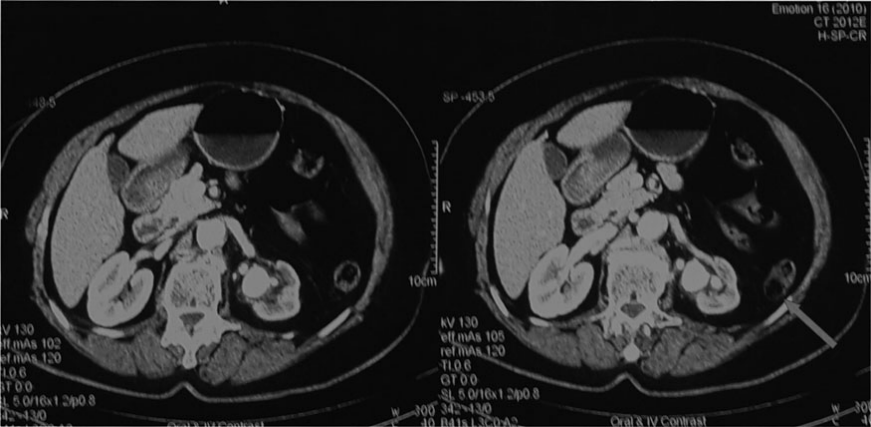
CT cross section slides showing large UPJ stone, thinning of left renal cortex, and renal pelvis dilation. *Red arrow* shows part of colon located beside left kidney (retro renal colon).

A 75-year-old woman underwent PCNL for partial staghorn stone in the left kidney, causing moderate hydronephrosis (Fig. 1). The procedure was performed by experienced faculty urologists under spinal anesthesia, using antibiotic prophylaxis. After retrograde catheterization with a 7F ureteral catheter in the lithotomy position, the patient was turned prone and percutaneous access was established under fluoroscopic guidance after contrast injection through the ureteral stent. Access to the collecting system was achieved by single puncture of posterior lower calix under C-arm X-ray guidance, the site of puncture was 1 cm below 12th rib and 3 cm medial to posterior axillary line to avoid colonic injury (CT scan shows partly retrorenal colon) (Fig. 2). After tract dilatation using coaxial serial Teflon-coated dilators, a 24F Amplatz sheath was placed and a slender nephroscope 17F (Karl Storz) was used for the procedure.

**FIG. 3.**
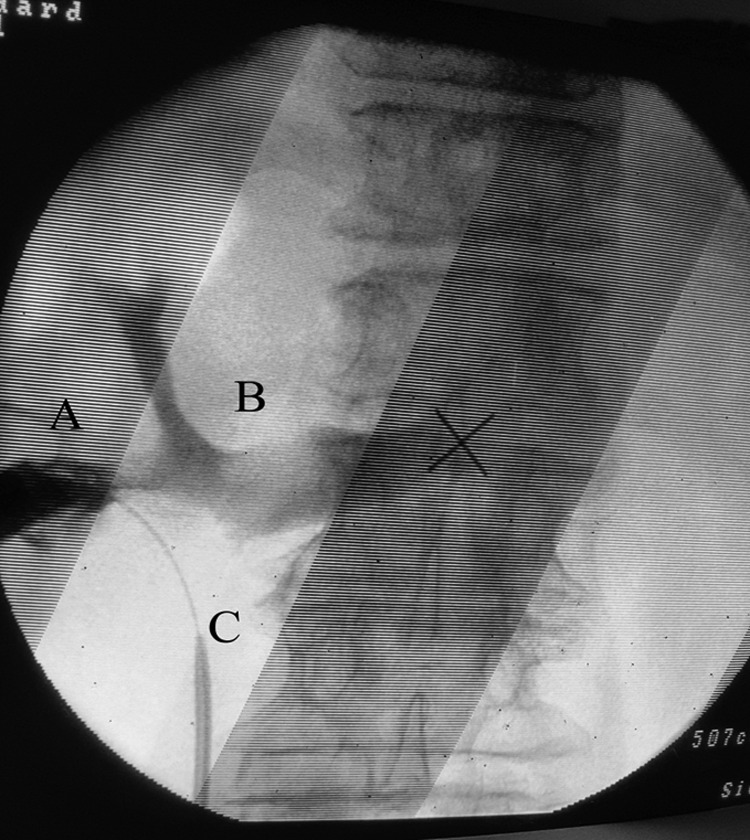
The figure shows contrast material left renal vein and both its tributaries. **(A)** Site of penetration of renal vein tributery during PCNL, **(B)** left main renal vein, **(C)** ureteral stent inside left ureter.

During inspection by nephroscope of the lower calix, there was intense venous bleeding from the injured part of the lower calix and we followed the tract through luminal structure and we suspected the possibility of injury to the structures surrounding the left kidney. Through the nephroscope, contrast material was injected to the abnormal tract, and the left renal vein with two tributaries and inferior vena cava were seen (Fig. 3), consequently direct injury and penetration to the left renal vein was confirmed.

Intraoperatively, the bleeding was controlled by placing the Amplatz sheath over the bleeding tract. The patient was hemodynamically stable throughout the whole procedure. The procedure continued and the stones were fragmented and removed; stone-free state was documented with C-arm and Double-J stent was inserted.

At the end of the procedure, 20F Foley catheter was inserted and used as a nephrostomy tube, its balloon was inflated with 3 mL fluid inside the renal pelvis and pulled back with light pressure to the lower calix to press the site of injury to the renal vein tributaries and then was closed for 24 hours.

The patient was kept in the ICU and she remained hemodynamically stable, with clear urine through both urethral and nephrostomy catheter; renal function and hemoglobin were normal. Antegrade pyelography was done on the second postoperative day; there was distally patent ureter with no extravasation, neither contrast leak to renal vein. At third postoperative day, urethral catheter was removed and the patient was discharged home, with nephrostomy catheter in place.

## Discussion

Puncturing and dilatation are two main steps in PCNL procedure, occasionally the puncturing needle or dilators pierce into the renal parenchyma and may migrate into the renal vein even to the vena cava. The proximity of the renal vein to the renal pelvis and major posterior calices predisposes them to injury during sequences of PCNL.^6^ Our patient had large UPJ stone extending to the lower calix. Concomitant infection and inflammation may have made the renal pelvicaliceal wall and surrounding structures more friable and susceptible to the injury.

The mechanism of injury could be puncturing too medially. Preoperative CT scanning shows that the colon was retrorenal in location (Fig. 3); therefore, the site of puncturing was decided to be more medially to avoid injury to the colon, by this we were near to the renal pedicle, consequently increasing the risk of vascular injury. In addition, overzealous dilatation and injury to the lower caliceal wall were other contributing factors.

Significant venous injuries during percutaneous renal surgery are most probably underdiagnosed.^7^ Since contrast material generally is not injected during percutaneous procedures to prevent extravasation and obscuring the fluoroscopic image, major injuries may be missed. Clotting of these venous injuries may occur during the operation and, therefore, the injury may not be evident on nephrostograms performed at the conclusion of the procedure or at follow-up.^8^

In our case, when we faced intense venous bleeding and an abnormal tract, injury to the renal vein tributary was confirmed by contrast injection through the nephroscope (Fig. 3).

At the end of the procedure, we tamponed the injured vein by using inflated Foley catheter. Pressure effect of the balloon is an effective mechanism for controlling venous bleeding, which is a routine practice after PCNL. Since venous bleeding can be controlled by optimal pressure effect from the balloon on the injured vessel and renal parenchymal tissue, because of less muscle content in the wall of the vein, there is minimal contraction during injury, in comparison with arterial wall contraction.^9^

Our case is one of the most uncommon cases in the literature regarding direct injury and penetration into the vascular system.^7,10,11^

The intraoperative recognition and diagnosis of renal vein injury are challenging. We recommend conservative management and follow-up, in a hemodynamically stable patient, with the availability of the next plan for angioembolization or other endovascular management, for direct bleeding from injured vein that is usually manifested by persistent hematuria or hematoma, or for other complications such as arteriovenous fistula, aneurism, or pseudoaneurism. In our case fortunately, there was no other complications.

## Conclusions

Renal vein injuries during PCNL can be associated with massive hemorrhage. Patients with major vascular injuries from percutaneous renal surgery can be treated conservatively using a nephrostomy catheter as a tamponade.

## Abbreviations Used

CT: computed tomography
ICU: intensive care unit
PCNL: percutaneous nephrolithotomy
UPJ: ureteropelvic junction
